# Does breast size matter? The thermoregulatory, perceptual and mechanical properties of the breast

**DOI:** 10.1113/EP092441

**Published:** 2025-03-14

**Authors:** Hannah Blount

**Affiliations:** ^1^ Skin Sensing Research Group, School of Health Sciences University of Southampton Southampton UK

**Keywords:** breast, exercise, female, morphology

## 1 INTRODUCTION

Connections link a sequence of three related research papers. The central article which links the other two papers has been published in *Experimental Physiology*. In a Connections article, an author (or authors) of the central article outlines its principal novel findings, tracing how they were influenced by the first article and how the central article has contributed to the developments made in the third article. The author(s) may also speculate on the direction of future research in the field. Connections articles aim to set the research in a wide context.

The size and shape of women's breasts can vary greatly between individuals and can change over time due to body mass, menstrual phases, pregnancy, breast feeding and menopause. However, most changes in breast size and shape occur during puberty, during which there is a large difference amongst women in the extent to which the breasts grow and consequently breast size. Variation in breast size will cause variation in the skin surface area across the breast, which could in turn impact the thermoregulatory, mechanical and perceptual properties of breast skin. However, the relationship between breast size and such changes in breast skin properties has until recently remained unclear. It is valuable to broaden our understanding of the impact of breast size on such parameters given over 85% of females consider sports bras essential to support and protect the breast and to reduce discomfort during exercise; yet the challenges associated with finding bras that are thermally comfortable and mechanically supportive (especially when saturated with sweat) and that accommodate individual variations in breast size can act as a barrier to exercise participation in women. A recent trilogy of studies (i.e., Blount et al., 2024a, 2024b, 2025) aimed to shed light on this apparently simple but multidimensional question; how and when does breast size matter when considering thermoregulatory, mechanical and perceptual properties of breast skin?

By integrating findings on sweat regulation, sensory perception and mechanical skin properties, these studies collectively aimed to address gaps in knowledge about female‐specific thermoregulation and sensation influenced by breast size. Broadening this fundamental physiological knowledge aimed to improve activewear design to mitigate barriers to exercise, enhance comfort and support an active lifestyle for women.

Using an experimental exercise trial to drive sweating, individual differences in breast surface area were observed to modulate both sweat gland density and local sweat rates in healthy young to middle‐aged females. As breast size increases, the density of sweat glands and local sweat rates decreased across the whole breast (Blount et al., 2024a). These findings confirm the previously established relationship between sweat gland density and whole‐body morphology, and they further extended this observation to the female breast, such that as skin surface area increases and stretches, the density of sweat glands decreases. Previous evidence in the literature would suggest that biophysical parameters, such as sweat gland density, often share similar patterns to neural receptor density, such that the perceptual sensitivities would mirror the sweating pattern across the breast with varying sizes. Breasts begin to grow during puberty, following nervous system development and after the point at which the number of sweat glands is set (i.e., age 2). Therefore, you could expect the relationship between breast size and sweat gland density and breast size and sensation to be similar. However, this was not consistently found to be the case in these studies. Variations of thermal sensitivity and wetness perception due to breast size appear to be skin site specific (i.e., above the nipple) and sensory stimuli dependent (i.e., warm sensitivity, but not cold or wetness sensitivity), rather than being a consistent feature of all the skin covering the female breast (Blount et al., 2025). This is such that as breast size increased, warm sensitivity decreased in the upper breast region only. Furthermore, irrespective of breast size, thermal and wetness sensitivity over the breast appeared to be relatively consistent, the exception being the skin sightly above the nipple, which presented a reduced cold sensitivity compared to other breast sites. From a skin mechanics perspective, the above nipple region was also found to be the main area of interest, as skin stiffness increased with increased breast size (Blount et al., 2024b). This is likely due to greater breast mass, and thus there are higher skin strains in larger breasted women. Yet no effect of breast size on skin stiffness was found in the lower breast region.

A consistent finding across these papers is that the upper breast region is an area whose thermoregulatory, perceptual and mechanical properties are regularly impacted by breast size. Yet, the extent to which these changes are related is open to question. It seems logical that a reduction in sweat gland density with increased breast size demonstrates an amount of skin stretch, which in turn is likely to cause increased skin strain and thus stiffness. This relationship was clearly observed in the upper breast region in our studies. Yet despite a size‐dependent relationship with sweat gland density in the lower breast region, we observed no size‐dependent relationship with skin stiffness in this area. We hypothesised that, despite being stretched with growth, the lower breast region is likely to be under lower mechanical load (i.e., it is required to bear less of the breast tissues load), which could in turn reduce any size‐dependent effect on skin stiffness. What remains somewhat ambiguous is the observation that thermal and wetness sensitivity did not consistently change with breast size. If a size‐dependent relationship existed with thermal and wetness sensitivity, one would expect to see this pattern across the whole breast, similar to the relationship between sweat gland density and breast surface area. However, given that only one breast region with one thermal stimulus reflected a relationship with breast size, we can infer that this is not a generalised feature of the breast. A plausible reason for this could be that sensory function is not a primary role of the breast. We hypothesise that the dispersion of thermoreceptors may be relatively sparse across the breast such that the ability to quantify sensation is more limited across this region compared to other body sites which are more thermally sensitive, such as the hands. Hence, we are less likely to see a breast size‐dependent effect.

**Connected Articles**

- Blount, H., Valenza, A., Ward, J., Caggiari, S., Worsley, P. R., & Filingeri, D. (2024). The effect of female breast surface area on heat‐activated sweat gland density and output. *Experimental Physiology*, *109*(8), 1330–1340.
- Blount, H., Valenza, A., Ward, J., Caggiari, S., Worsley, P. R., & Filingeri, D. (2024). The effect of female breast surface area on skin stiffness and tactile sensitivity at rest and following exercise in the heat. *Experimental Physiology*, *109*(10), 1698–1709.
- Blount, H., Valenza, A., Ward, J., Caggiari, S., Worsley, P. R., & Filingeri, D. (2025). The effect of female breast surface area on cutaneous thermal sensation, wetness perception and epidermal properties. *Experimental Physiology*, *110*(2), 248–260.

When considering the question ‘does breast size matter?’, in light of our collective findings, the answer appears to be ‘yes’ in relation to the sweating apparatus and the skin mechanical properties of the upper breast region only; however, the answer may also be ‘no’, in the case of breast thermal and wetness perceptions at rest.

These initial, fundamental findings have increased our understanding of the impact of breast size on skin properties, and this knowledge could inform tailored considerations in sportswear design, targeting specific regions of the breast to optimise comfort and function from a thermal and mechanical perspective for women of varying breast sizes. For example, bra design may consider the need to reduce strains and stiffness in the upper breast region, more so for larger breasted women; it may also consider the impact of higher local sweat rates in smaller breasted women which could lead to greater bra saturation. However, as it is often the case, these findings have also opened further fundamental and applied questions, which we hope future research will address. For example: when performing exercise, does the impact of size‐dependent sweat rates and skin stiffness translate into different perceptual outcomes from those we observed at rest? And if so, how does this further influence our approach to improving the design of sportswear to account for individual variability in breast size?

## AUTHOR CONTRIBUTIONS

Sole author.

## CONFLICT OF INTEREST

None declared.
